# Serum polyunsaturated fatty acids and hearing threshold shifts in adults in the United States: A cross-sectional study

**DOI:** 10.3389/fpubh.2022.939827

**Published:** 2022-11-16

**Authors:** Lili Long, Zhenchao Jia, Xinghua Tang

**Affiliations:** ^1^Department of Otorhinolaryngology, Sichuan University Hospital of Sichuan University, Chengdu, China; ^2^Department of Prevention and Health Care, Sichuan University Hospital of Sichuan University, Chengdu, China; ^3^Department of Otorhinolaryngology Head and Neck Surgery, Sichuan Provincial People's Hospital, University of Electronic Science and Technology of China, Chengdu, China

**Keywords:** polyunsaturated fatty acids, hearing threshold shift, National Health and Nutrition Examination Survey, cross-sectional study, adults

## Abstract

**Background:**

Few studies have evaluated the association between polyunsaturated fatty acids (PUFAs) and hearing levels. This study aimed to investigate the association between serum PUFAs and hearing threshold shifts in US adults.

**Methods:**

We investigated 913 adults from the National Health and Nutrition Examination Survey (NHANES) 2011–2012. Multivariate linear regression analyses were conducted to evaluate associations between PUFA and hearing threshold shifts.

**Results:**

Overall, 11 serum PUFAs were inversely associated with low-frequency thresholds, especially in men, and were positively related to high-frequency thresholds, particularly in the 40–59 years old cohort. Furthermore, some serum PUFAs were positively associated with both hearing threshold subgroups in women.

**Conclusion:**

Some PUFAs tend to be beneficial for low-frequency hearing status and detrimental to the high-frequency hearing threshold. The male sex may play a protective role in this association, while the female sex and middle age may be detrimental in the effect of PUFAs on hearing function.

## Introduction

Hearing loss (HL) is the most common sensory deficit in humans. More than 30 million adults in the United States, nearly 15% of the total population, have some degree of HL (1). Hearing impairment adversely affects social engagement and is associated with impaired quality of life, dementia, depression, and increased mortality (2–4). The estimated direct and indirect medical costs resulting from hearing impairment have increased from $3.3 million to 12.8 million annually in the United States (5). This health burden is escalating; hence, studying risk factors to develop preventive and therapeutic strategies is essential to reduce the effect and burden of hearing impairment.

The cochlea in the inner ear is highly vascularized and is supplied by a single feed artery (6). It is assumed that impaired inner ear perfusion and ischemic vascular damage of the cochlea can cause hearing impairment (7). Cardiovascular disease events (e.g., myocardial infarction, ischemic heart disease, and stroke) showed a moderate association with hearing impairment in a cohort study (8). Previous studies have further reported on the relationship between polyunsaturated fatty acids (PUFAs) and many diseases (9). n-3 PUFAs have been shown to exert protective effects against cardiovascular diseases, such as heart failure and stroke (9, 10). Hence, it is plausible that PUFAs may also play an important cochlear protective role for the auditory system.

To date, there have been only a few population-based studies investigating the association between PUFAs and the risk of hearing impairment (11–15), three of which studied the effect of total dietary PUFA intake, particularly n-3 PUFAs on low-frequency or speech-frequency hearing impairment (11, 12, 14). Two studies examined the relationship between plasma PUFAs and hearing status but only in old or young people (13, 15). Therefore, we performed this study using data from the National Health and Nutrition Examination Survey (NHANES) database to investigate whether cross-sectional associations exist between individual serum PUFAs and both low-, and high-frequency hearing threshold shifts in adults aged 20–69 years in the United States.

## Methods

### Ethics statement

This study utilized publicly accessible data from the NHANES website (https://www.cdc.gov/nchs/nhanes/Index.htm). The NHANES data were approved by the National Center for Health Statistics Institutional Review Board in accordance with the Declaration of Helsinki. Informed consent was obtained from all the eligible subjects.

### Study population

**Figure 1:**
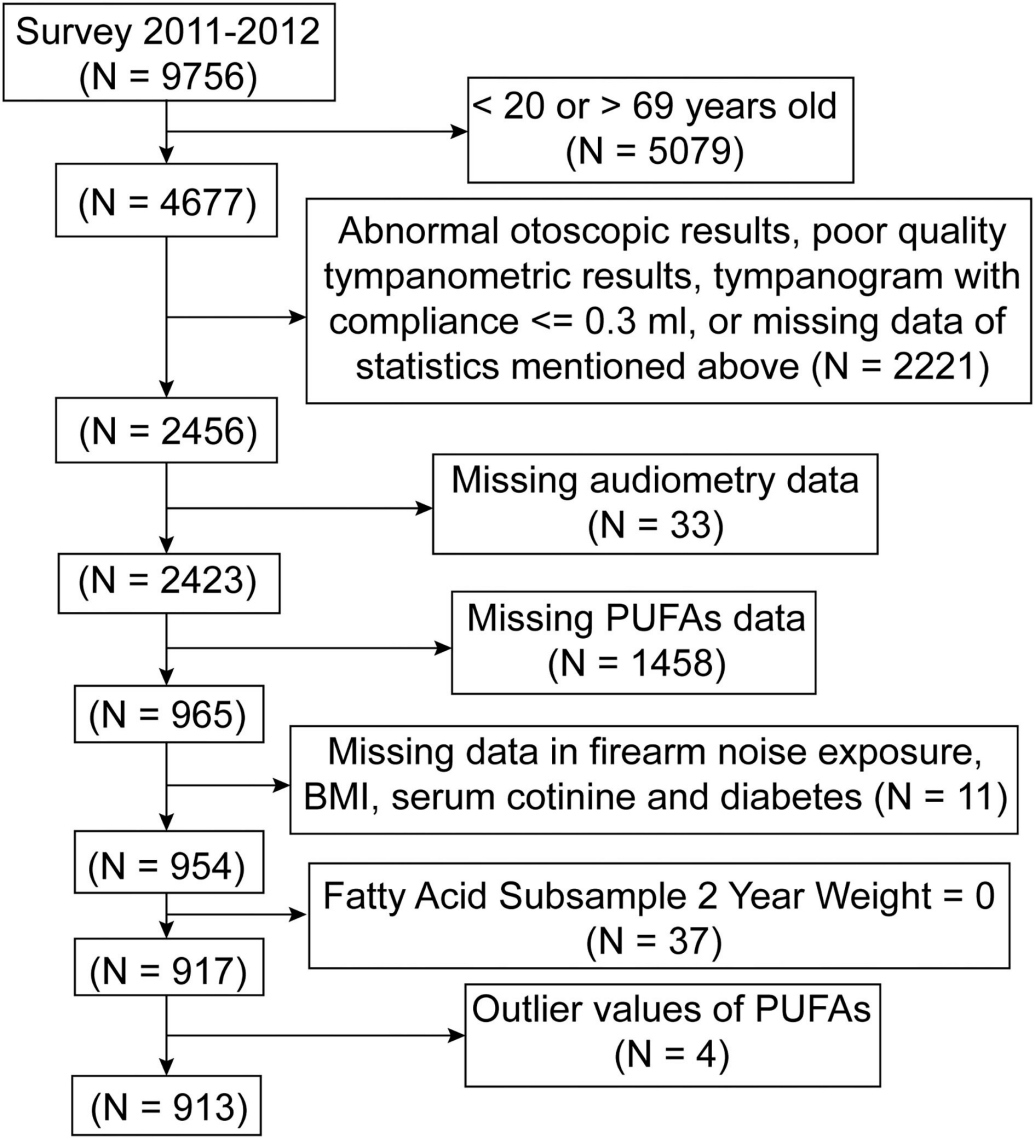
Flow chart of the patient selection process. NHANES, National Health and Nutrition Examination Survey.

The National Health and Nutrition Examination Survey is a national survey conducted every year by the National Center for Health Statistics (NCHS) of the Centers for Disease Control and Prevention. The survey is combined with a series of physical examinations, interviews, and laboratory tests and uses a complex, multistage, probability sample design to be representative of the civilian, noninstitutionalized US population. Cross-sectional data examined in this study were collected from participants enrolled in the 2011–2012 cycle of the NHANES, as this is the only cycle containing results of serum fatty acid tests. The complete selection procedure for the study is shown in Figure 1. Audiometry examinations were conducted in adults aged 20–69 years. Participants lacking complete data on the otoscopic test, tympanogram test, audiometry test, and PUFAs measurement or with missing covariate data were excluded, as were participants with abnormal otoscopic results, poor-quality tympanogram results, or tympanogram with compliance ≤ 0.3 ml. Participants with the subsample weight value assigned as “0” in their records were excluded, as they did not provide blood specimens. Four participants with outlier values of PUFAs were also excluded. Finally, 913 adults were included in the study.

### Blood PUFAs measurement

Serum samples were processed, stored, and shipped to the Division of Laboratory Sciences, National Center for Environmental Health, Centers for Disease Control and Prevention, Atlanta, GA for testing. Fasting serum fatty acid concentrations were measured by electron capture negative-ion mass spectrometry based on a modification of the method outlined by Lagerstedt et al. (16). More details regarding the PUFA quantification procedure and analytical methods are available on the NHANES website (https://wwwn.cdc.gov/Nchs/Nhanes/2011-2012/FAS_G.htm#LBXED1).

### Audiometric measurement

Standardized pure-tone air conduction audiometric measurements were conducted in a dedicated sound-isolated room by a trained examiner. Hearing thresholds were tested on both ears of the participants at frequencies between 500 and 8,000 Hz. Pure-tone average (PTA) hearing thresholds were calculated at low (0.5, 1, and 2 kHz) and high (4, 6, and 8 kHz) frequencies. More details about the audiometry procedure and analytical methods are available on the NHANES website (https://wwwn.cdc.gov/Nchs/Nhanes/2011-2012/AUX_G.htm).

### Covariates

Potential covariates considered in the analyses included age, sex, race/ethnicity, education level, body mass index (BMI), diabetes, hypertension, serum cotinine, firearm noise exposure, occupational noise exposure, and recreational noise exposure. Information on age, sex, race/ethnicity, education level, diabetes, hypertension, and noise exposure was obtained from the in-home self-reported questionnaire. BMI data were calculated from the weight and height data recorded during the physical examination. Diabetes was defined as “other than during pregnancy, ever been told by a doctor or health professional had diabetes or sugar diabetes.” The answer of “borderline” was also considered as diabetes (17). Hypertension was defined as “ever been told by a doctor or other health professional had hypertension, also called high blood pressure” (17). Firearm noise exposure was defined as “ever used firearms for any reason,” occupational noise exposure was defined as “ever had a job or combination of jobs exposed to loud sounds or noise for 4 or more hours a day, several days a week,” and recreational noise exposure was defined as “ever been exposed to very loud noise or music for 10 or more hours a week” (17).

### Statistical analysis

The study used Fatty Acid Subsample 2 Year Weight of the 2011–2012 NHANES cycle to estimate representative measures for the United States population, following the guidelines of the NCHS (18, 19). Serum concentrations of saturated, monounsaturated, and polyunsaturated fatty acids were measured in the 2011–2012 NHANES survey that included a total of 11 serum n-3 and n-6 PUFAs, which were used for analyses in our study. These PUFAs were linoleic acid (LA, 18:2n-6), γ-linolenic acid (GLA, 18:3n-6), eicosadienoic acid (EDA, 20:2n-6), homo-γ-linolenic acid (HGLA, 20:3n-6), arachidonic acid (AA, 20:4n-6), docosatetraenoic acid (DTA, 22:4n-6), docosapentaenoic acid (DPAn-6, 22:5n-6), α-linolenic acid (ALA, 18:3n-3), eicosapentaenoic acid (EPA, 20:5n-3), docosapentaenoic acid (DPA, 22:5n-3), and docosahexaenoic acid (DHA, 22:6n-3). Weighted statistical differences in demographic and potential hearing-related covariables between samples grouped by sex were evaluated (Table 1). Categorical data were shown as percentages, and continuous data were presented as mean ± standard deviation (SD). The total number of participants (*N* = 913) was divided into tertiles for each PUFA, from the lowest concentration of each PUFA to the highest level, with almost the same number of subjects in each tertile (33%). The range of PUFA values for each tertile is shown in Supplementary Table S1. Multivariate linear regression analysis was performed to determine regression coefficients (β) and 95% confidence intervals (CIs) between PUFAs and hearing threshold shifts, adjusting for potential confounders, including age, sex, race/ethnicity, education level, BMI (categorical), diabetes, hypertension, serum cotinine, firearm noise exposure, occupational noise exposure, and recreational noise exposure. Tests for a linear trend across tertiles of serum PUFAs were conducted using the median serum PUFAs in each tertile as a continuous variable. The interactions of PUFAs with age and sex in influencing hearing thresholds were evaluated. Multivariate linear regression analysis stratified by age and sex was performed. All statistical analyses were conducted using the R programming language (version 3.6.1). A *p*-value of less than 0.05 was considered statistically significant.

**Table 1:** Weighted demographic characteristics of the study participants.

**Variables**	**Male (*N* = 474)**	**Female (*N* = 439)**	***P*-value^a^**
**Continuous variables, mean** **±SD**			
Age (years)	41.68 ± 13.75	42.35 ± 13.95	0.4692
BMI (kg/m^2^)	28.89 ± 5.80	29.28 ± 7.39	0.3792
Low-frequency PTA (dB)^b^	7.37 ± 7.65	7.50 ± 7.75	0.7876
High-frequency PTA (dB)^b^	21.82 ± 18.70	16.20 ± 12.83	**<0.0001**
**Categorical variables, %**			
**Race/Ethnicity**			0.3074
Mexican American	7.08	8.93	
Non-Hispanic White	69.13	64.20	
Non-Hispanic Black	10.02	13.02	
Other races	13.77	13.84	
**Education level**			**0.0024**
Below high school	13.86	12.85	
High school	21.39	13.08	
Above high school	64.75	74.06	
**BMI (categorical)**			**0.0208**
Underweight (<18.5 kg/m^2^)	0.53	2.15	
Normal (≥18.5 kg/m^2^, <25 kg/m^2^)	25.45	30.00	
Overweight (≥25 kg/m^2^, <30 kg/m^2^)	37.04	29.97	
Obesity (≥30 kg/m^2^)	36.97	37.87	
Diabetes	7.19	7.72	0.7636
Hypertension	27.27	25.39	0.5195
Serum cotinine (≥10 ng/ml)	31.92	18.52	**<0.0001**
Firearm noise exposure	60.76	29.76	**<0.0001**
Occupational noise exposure	44.11	23.32	**<0.0001**
Recreational noise exposure	17.80	7.71	**<0.0001**

BMI, body mass index; PTA, pure-tone average. ^a^*p*-values of continuous variables and categorical variables were calculated by the weighted linear regression model and weighted chi-square test, respectively. ^b^Low-frequency and high-frequency PTA values in the better ear were computed from the average hearing thresholds of 0.5, 1, and 2 kHz and 4, 6, and 8 kHz, respectively.

The bold values indicate the significant values.

## Results

### Characteristics of the study participants

The study sample included 913 participants that included 439 women (weighted mean, 42.35 ± 13.95 years) and 474 men (weighted mean, 41.68 ± 13.75 years) aged between 20 and 69 years, sampled from the US population. The means ± SD of low-frequency and high-frequency PTA hearing thresholds were 7.37 ± 7.65 and 21.82 ± 18.70 dB, in male participants, respectively, and 7.50 ± 7.75 and 16.20 ± 12.83 dB in female subjects, respectively. The average high-frequency hearing status of men was worse than that of women. The education levels of men were lower than those of women. The BMI of men was higher than that of women, and men were more likely to be overweight than women. The level of serum cotinine, the biomarker of passive and positive smoking exposure, was higher in men than that in women. Men were exposed to more firearm noise, occupational noise, and recreational noise than women (all *p* < 0.05).

### Multivariate regression analysis: Association between PUFAs and hearing thresholds

Table 2 shows the associations between 11 individual PUFAs with low-frequency and high-frequency hearing thresholds using a multivariate linear regression model. All PUFAs were converted to a categorical variable (tertiles) and were used as a continuous variable to calculate the linear trend. In the unadjusted model (crude model), the *p*-value for trend indicates that almost all 11 PUFAs were positively associated with low-frequency and high-frequency PTA hearing threshold shifts. Only the associations of LA, AA, DPAn-6, ALA, and DHA with low-frequency PTA were not significant. In the fully adjusted model (model 2), AA and DHA were inversely related to low-frequency PTA, while EDA, HGLA, and DPAn-6 showed positive associations with high-frequency PTA. However, significant *p* for trend was not observed among the tertiles of 6 other PUFAs and hearing threshold shifts (all *p* for trend ≥ 0.05).

**Table 2:** Multivariable linear regression model of outcomes of hearing thresholds.

**Variables (umol/L)**			**Low-frequency PTA (dB)**			**High-frequency PTA (dB)**		
			**Crude model**	**Model 1**	**Model 2**	**Crude model**	**Model 1**	**Model 2**
n-6	LA	β (95% CI)	0.52 (−0.10, 1.15)	−0.24 (−0.81, 0.32)	−0.18 (−0.76, 0.40)	2.91 (1.58, 4.24)	0.89 (−0.16, 1.94)	0.92 (−0.15, 1.98)
		*P*_trend_	0.1026	0.3987	0.5472	**<0.0001**	0.0978	0.0928
	GLA	β (95% CI)	1.36 (0.75, 1.97)	0.06 (−0.52, 0.63)	−0.01 (−0.60, 0.58)	4.62 (3.35, 5.89)	0.67 (−0.40, 1.73)	0.51 (−0.57, 1.59)
		*P*_trend_	**<0.0001**	0.8487	0.9697	**<0.0001**	0.2192	0.3584
	EDA	β (95% CI)	0.84 (0.24, 1.45)	0.01 (−0.54, 0.56)	−0.07 (−0.64, 0.50)	4.37 (3.11, 5.64)	2.18 (1.17, 3.19)	2.22 (1.19, 3.26)
		*P*_trend_	**0.0064**	0.9813	0.8216	**<0.0001**	**<0.0001**	**<0.0001**
	HGLA	β (95% CI)	0.90 (0.28, 1.53)	0.14 (−0.43, 0.71)	0.03 (−0.57, 0.62)	3.49 (2.17, 4.81)	1.57 (0.52, 2.61)	1.35 (0.27, 2.44)
		*P*_trend_	**0.0047**	0.6290	0.9318	**<0.0001**	**0.0033**	**0.0148**
	AA	β (95% CI)	0.46 (−0.15, 1.08)	−0.82 (−1.39, −0.26)	−0.80 (−1.37, −0.22)	3.32 (2.03, 4.61)	−0.21 (−1.26, 0.85)	−0.18 (−1.24, 0.89)
		*P*_trend_	0.1378	**0.0045**	**0.0070**	**<0.0001**	0.6989	0.7459
	DTA	β (95% CI)	0.74 (0.12, 1.36)	0.02 (−0.54, 0.58)	−0.16 (−0.75, 0.43)	2.96 (1.65, 4.28)	0.64 (−0.40, 1.68)	0.30 (−0.78, 1.39)
		*P*_trend_	**0.0192**	0.9485	0.5895	**<0.0001**	0.2293	0.5808
	DPAn−6	β (95% CI)	0.42 (−0.20, 1.03)	0.12 (−0.43, 0.67)	−0.04 (−0.60, 0.53)	2.07 (0.76, 3.38)	1.42 (0.40, 2.44)	1.22 (0.19, 2.26)
		*P*_trend_	0.1868	0.6741	0.8938	**0.0021**	**0.0064**	**0.0206**
n-3	ALA	β (95% CI)	0.53 (−0.10, 1.15)	0.16 (−0.40, 0.72)	0.17 (−0.41, 0.74)	2.17 (0.84, 3.49)	1.00 (−0.03, 2.03)	1.00 (−0.06, 2.05)
		*P*_trend_	0.0977	0.5711	0.5694	**0.0014**	0.0580	0.0637
	EPA	β (95% CI)	0.94 (0.33, 1.55)	−0.52 (−1.10, 0.05)	−0.37 (−0.96, 0.22)	4.57 (3.30, 5.85)	0.61 (−0.46, 1.67)	0.95 (−0.13, 2.03)
		*P*_trend_	**0.0025**	0.0756	0.2137	**<0.0001**	0.2639	0.0863
	DPA	β (95% CI)	1.45 (0.83, 2.07)	−0.23 (−0.83, 0.37)	−0.25 (−0.86, 0.37)	4.54 (3.24, 5.84)	−0.32 (−1.44, 0.79)	−0.41 (−1.53, 0.71)
		*P*_trend_	**<0.0001**	0.4538	0.4317	**<0.0001**	0.5693	0.4748
	DHA	β (95% CI)	0.01 (−0.60, 0.62)	−0.91 (−1.46, −0.36)	−0.79 (−1.36, −0.23)	2.26 (0.97, 3.55)	0.28 (−0.75, 1.31)	0.87 (−0.17, 1.92)
		*P*_trend_	0.9831	**0.0013**	**0.0061**	**0.0006**	0.5979	0.1008

Crude Model = unadjusted. Model 1 = Crude Model + sex, age. Model 2 = Model 1 + race/ethnicity, education level, firearm noise exposure, occupational noise exposure, recreational noise exposure, serum cotinine, BMI, diabetes, and hypertension.

The bold values indicate the significant values.

### Multivariate regression analysis stratified by age: Association between PUFAs and hearing thresholds

Table 3 and Supplementary Table S2 show the results for 11 PUFAs in analyses stratified by age. In general, 5 PUFAs, HGLA, DTA, DPAn-6, EPA, and DHA were associated with hearing threshold shifts that differed by age (Table 3). People aged 40–59 years in the highest tertile of HGLA, DTA, DPAn-6, EPA, and DHA and people aged 20–39 years in the highest tertile of HGLA had higher high-frequency PTA as compared to those in the lowest tertile of these PUFAs after adjusting for age, sex, race/ethnicity, education level, BMI, diabetes, hypertension, serum cotinine level, firearm noise exposure, occupational noise exposure, and recreational noise exposure (β = 2.03, 4.38, 5.90, 6.09, 6.27, and 2.03, *p* for trend = 0.0457, 0.0216, 0.0020, 0.0017, 0.0014, and 0.0457, respectively, all *p* for interaction <0.05; Table 3). However, DTA demonstrated an inverse trend with high-frequency PTA in subjects aged 60–69 years (β = −10.76, *p* for trend = 0.0020, *p* for interaction = 0.0371; Table 3). LA, EDA, and ALA showed no statistically significant interactions with age on the prediction of hearing threshold shifts (Supplementary Table S2).

**Table 3:** Adjusted^a^ associations between PUFAs and hearing threshold shifts stratified by age (*N* = 913).

					***P*_trend_**	***P*_interaction_**
		**HGLA (umol/L)** β **(95% CI)**				
		**Tertile 1**	**Tertile 2**	**Tertile 3**		
**Low-frequency PTA**	20 ≤ y <40	Ref	0.11 (−1.06, 1.27)	0.77 (−0.52, 2.05)	0.2502	**0.0041**
	40 ≤ y <60	Ref	−0.58 (−2.56, 1.40)	0.13 (−1.77, 2.02)	0.7199	
	60 ≤ y <69	Ref	−1.83 (−7.60, 3.94)	−3.63 (−9.03, 1.78)	0.1846	
**High-frequency PTA**	20 ≤ y <40	Ref	−0.01 (−1.76, 1.73)	2.03 (0.10, 3.95)	**0.0457**	**0.0182**
	40 ≤ y <60	Ref	5.49 (1.21, 9.78)	6.38 (2.28, 10.48)	**0.0056**	
	60 ≤ y <69	Ref	−3.72 (−12.10, 4.65)	−3.83 (−11.67, 4.02)	0.3737	
		**DTA (umol/L)** β **(95% CI)**				
		**Tertile 1**	**Tertile 2**	**Tertile 3**		
**Low-frequency PTA**	20 ≤ y <40	Ref	−0.31 (−1.46, 0.84)	0.58 (−0.73, 1.89)	0.4546	0.2038
	40 ≤ y <60	Ref	1.09 (−0.84, 3.02)	0.71 (−1.19, 2.60)	0.5854	
	60 ≤ y <69	Ref	−6.55 (−11.71, −1.39)	−4.46 (−9.03, 0.10)	0.1000	
**High-frequency PTA**	20 ≤ y <40	Ref	0.04 (−1.70, 1.78)	1.19 (−0.79, 3.16)	0.2698	**0.0371**
	40 ≤ y <60	Ref	0.80 (−3.40, 4.99)	4.38 (0.26, 8.50)	**0.0216**	
	60 ≤ y <69	Ref	−8.22 (−15.56, −0.87)	−10.76 (−17.25, 4.26)	**0.0020**	
		**DPAn**−**6 (umol/L)** β **(95% CI)**				
		**Tertile 1**	**Tertile 2**	**Tertile 3**		
**Low-frequency PTA**	20 ≤ y <40	Ref	1.03 (−0.72, 2.78)	0.42 (−1.33, 2.18)	0.9167	0.8037
	40 ≤ y <60	Ref	1.03 (−0.72, 2.78)	0.42 (−1.33, 2.18)	0.6942	
	60 ≤ y <69	Ref	−0.96 (−6.03, 4.11)	−0.77 (−5.27, 3.72)	0.7439	
**High-frequency PTA**	20 ≤ y <40	Ref	1.73 (−2.05, 5.52)	5.90 (2.11, 9.69)	0.0676	**0.0010**
	40 ≤ y <60	Ref	1.73 (−2.05, 5.52)	5.90 (2.11, 9.69)	**0.0020**	
	60 ≤ y <69	Ref	−0.24 (−7.45, 6.97)	−5.93 (−12.31, 0.46)	0.0648	
		**EPA (umol/L)** β **(95% CI)**				
		**Tertile 1**	**Tertile 2**	**Tertile 3**		
**Low-frequency PTA**	20 ≤ y <40	Ref	−0.92 (−2.05, 0.22)	−1.14 (−2.42, 0.14)	0.0628	0.4376
	40 ≤ y <60	Ref	0.48 (−1.50, 2.46)	−0.64 (−2.58, 1.30)	0.3733	
	60 ≤ y <69	Ref	−2.66 (−8.24, 2.92)	0.63 (−4.54, 5.81)	0.6878	
**High-frequency PTA**	20 ≤ y <40	Ref	0.99 (−0.72, 2.71)	−0.07 (−2.00, 1.87)	0.9011	**0.0334**
	40 ≤ y <60	Ref	1.22 (−3.06, 5.50)	6.09 (1.90, 10.28)	**0.0017**	
	60 ≤ y <69	Ref	−10.50 (−18.31, −2.69)	−0.37 (−7.61, 6.88)	0.7716	
		**DHA (umol/L)** β **(95% CI)**				
		**Tertile 1**	**Tertile 2**	**Tertile 3**		
**Low-frequency PTA**	20 ≤ y <40	Ref	−1.26 (−2.39, −0.13)	−1.78 (−3.07, −0.48)	**0.0048**	0.3053
	40 ≤ y <60	Ref	−0.99 (−2.73, 0.76)	−0.94 (−2.72, 0.84)	0.3134	
	60 ≤ y <69	Ref	−0.15 (−5.63, 5.33)	−1.92 (−6.33, 2.49)	0.3878	
**High-frequency PTA**	20 ≤ y <40	Ref	−0.83 (−2.56, 0.89)	−0.55 (−2.52, 1.43)	0.5064	**0.0028**
	40 ≤ y <60	Ref	2.01 (−1.77, 5.78)	6.27 (2.42, 10.12)	**0.0014**	
	60 ≤ y <69	Ref	−8.00 (−15.82, 0.18)	−5.93 (−12.31, 0.46)	0.4079	

^a^Adjusted for age, sex, race/ethnicity, education level, BMI, diabetes, hypertension, serum cotinine, firearm noise exposure, occupational noise exposure, and recreational noise exposure.

The bold values indicate the significant values.

### Multivariate regression analysis stratified by sex: Association between PUFAs and hearing thresholds

Table 4 and Supplementary Table S3 show the associations of the tertiles of 11 PUFAs with different groups of hearing threshold shifts stratified by sex. Men in the highest tertile of EDA, AA, and DHA had better low-frequency hearing levels as compared to those in the lowest tertile after adjusting for age, race/ethnicity, education level, BMI, diabetes, hypertension, serum cotinine level, firearm noise exposure, occupational noise exposure, and recreational noise exposure (β = −1.75, −2.69, and −2.31, *p* for trend = 0.0386, 0.0008, and 0.0057, *p* for interaction = 0.0004, 0.0230, and 0.0313, respectively; Table 4). In contrast, women in the highest tertile of EDA and DPAn-6 had worse low-frequency hearing levels as compared to those in the lowest tertile after adjusting for confounders (β = 1.94 and 2.55, *p* for trend = 0.0168 and 0.0307, *p* for interaction = 0.0004 and 0.0027, respectively), so as ALA with high-frequency PTA (β = 4.13, *p* for trend = 0.0028, *p* for interaction = 0.0016) in women (Table 4). Although 9 out of 11 PUFAs showed statistically significant interactions with sex for the prediction of hearing threshold shifts, most showed no statistically significant relationship with hearing threshold shifts, except the 4 PUFAs mentioned above (Table 4 and Supplementary Table S3).

**Table 4:** Adjusted^a^ associations between PUFAs and hearing threshold shifts stratified by sex (*N* = 913).

					***P*_trend_**	***P*_interaction_**
		**EDA (umol/L)** β **(95% CI)**				
		**Tertile 1**	**Tertile 2**	**Tertile 3**		
**Low-frequency PTA**	Male	Ref	0.42 (−1.13, 1.98)	−1.75 (−3.37, −0.13)	**0.0386**	**0.0004**
	Female	Ref	0.77 (−0.81, 2.36)	1.94 (0.34, 3.54)	**0.0168**	
**High-frequency PTA**	Male	Ref	1.42 (−1.78, 4.62)	3.34 (0.01, 6.68)	0.0501	0.1601
	Female	Ref	1.36 (−1.00, 3.71)	5.41 (3.03, 7.78)	<0.0001	
		**AA (umol/L)** β **(95% CI)**				
		**Tertile 1**	**Tertile 2**	**Tertile 3**		
**Low-frequency PTA**	Male	Ref	−2.05 (−3.58, −0.51)	−2.69 (−4.24, −1.14)	**0.0008**	**0.0230**
	Female	Ref	0.48 (−1.10, 2.06)	−0.06 (−1.78, 1.67)	0.9547	
**High-frequency PTA**	Male	Ref	−1.89 (−5.07, 1.30)	−0.56 (−3.78, 2.66)	0.7411	0.2258
	Female	Ref	1.21 (−1.18, 3.59)	0.74 (−1.87, 3.35)	0.5723	
		**DPAn-6 (umol/L)** β **(95% CI)**				
		**Tertile 1**	**Tertile 2**	**Tertile 3**		
**Low-frequency PTA**	Male	Ref	0.20 (−1.55, 1.96)	−1.26 (−3.37, 0.84)	0.2321	**0.0027**
	Female	Ref	1.40 (−0.39, 3.18)	2.55 (0.24, 4.85)	**0.0307**	
**High-frequency PTA**	Male	Ref	2.13 (−1.46, 5.73)	4.16 (−0.16, 8.48)	0.0594	0.8201
	Female	Ref	2.22 (−0.46, 4.90)	3.54 (0.08, 7.01)	**0.0450**	
		**ALA (umol/L)** β **(95% CI)**				
		**Tertile 1**	**Tertile 2**	**Tertile 3**		
**Low-frequency PTA**	Male	Ref	0.96 (−0.67, 2.58)	−0.36 (−2.05, 1.33)	0.6071	**0.0002**
	Female	Ref	0.86 (−0.70, 2.42)	1.51 (−0.28, 3.30)	0.0959	
**High-frequency PTA**	Male	Ref	−2.07 (−5.40, 1.27)	0.66 (−2.80, 4.13)	0.6347	**0.0016**
	Female	Ref	1.12 (−1.20, 3.45)	4.13 (1.47, 6.79)	**0.0028**	
		**DHA (umol/L)** β **(95% CI)**				
		**Tertile 1**	**Tertile 2**	**Tertile 3**		
**Low-frequency PTA**	Male	Ref	−0.59 (−2.07, 0.89)	−2.31 (−3.94, −0.67)	**0.0057**	**0.0313**
	Female	Ref	−1.23 (−2.85, 0.39)	−1.54 (−3.22, 0.15)	0.5176	
**High-frequency PTA**	Male	Ref	−1.61 (−4.67, 1.44)	1.81 (−1.56, 5.18)	0.1018	0.3300
	Female	Ref	1.16 (−1.28, 3.60)	0.16 (−2.37, 2.70)	0.4506	

^a^Adjusted for age, race/ethnicity, education level, BMI, diabetes, hypertension, serum cotinine level, firearm noise exposure, occupational noise exposure, and recreational noise exposure.

The bold values indicate the significant values.

## Discussion

In this nationwide cross-sectional study, we identified a relationship between n-6, n-3 PUFAs and hearing threshold shifts of adults in the United States. This research indicated that some serum PUFAs were inversely associated with low-frequency PTA, especially in men, and were positively related to high-frequency PTA, particularly in the 40–59 years old cohort. Furthermore, some serum PUFAs were found to be positively associated with both hearing threshold subgroups in women after adjusting for confounders (Tables 2–4, Supplementary Tables S1, S2). To the best of our knowledge, this is the first cross-sectional study to investigate the relationship between individual serum PUFAs and hearing threshold shifts of adults in the United States. The findings of this study suggest that PUFAs may exert both beneficial and detrimental effects on human hearing status.

Three previous population-based studies found that higher increases in n-3 PUFAs were associated with reduced HL. HL was estimated using pure-tone audiometry at speech frequency (500, 1,000, 2,000, and 4,000 Hz) or by self-report (11, 12, 14). The study conducted by Dullemeijer et al. (13) testing plasma n-3 PUFAs showed an inverse association between n-3 PUFAs and low-frequency hearing levels, which were consistent with the results of a prior study (13). However, in a recent longitudinal observational cohort study, measuring plasma concentrations of n-3 and n-6 PUFAs, no clear link was found between PUFAs and hearing function (15).

The results of our study showed that AA and DHA were inversely associated with low-frequency PTA after adjusting for related cofounders and that EDA and DHA were inversely related to low-frequency PTA in men. The benefit of PUFAs on low-frequency hearing levels was almost consistent with findings of previous population-based and animal studies (11–14, 20). Cochlear blood flow must be well regulated to meet the metabolic demand of the inner ear. Impaired cochlear blood flow may lead to damage to hair cells, resulting in the development of hearing impairment. The n-3 PUFAs may benefit hearing by the maintenance of adequate cochlear vascular supply through multiple mechanisms, including triglyceride lowering, hypolipidemic properties, and anti-inflammatory and anti-atherothrombotic properties (21, 22). Evidence has also shown that dietary n-6 PUFA may help to improve endothelial function and chronic inflammation (23).

Though in general, some PUFAs were found to be beneficial for the low-frequency hearing threshold, and EDA, HGLA, and DPAn-6 showed a positive association with high-frequency PTA. DTA, DPAn-6, EPA, and DHA in participants aged 40–59 years and HGLA in participants aged 20–59 years were positively associated with high-frequency PTA. EDA and DPAn-6 were positively associated with low-frequency PTA in women, and ALA was associated with high-frequency PTA in women. There are some pieces of evidence to show the detrimental effect of PUFAs on the hearing status and auditory development, which support our findings (20, 24–28). The association of PUFAs with hearing level is more obvious in middle-aged participants younger than 60 years, indicating that the onset of PUFAs' effect on age-related hearing impairment is much earlier than that previously reported (11, 13). PUFAs showed a protective role for hearing in men and a detrimental role in women, which is in contrast with previous findings (12, 14). More studies are needed to better understand the differences between men and women to reach a consensus.

Our study has several strengths, including the large and nationally representative sample cohort extracted from the NHANES. The selection was standardized to achieve minimized selection bias. Furthermore, standardized, audiometric testing was used to measure the pure-tone hearing threshold. Participants with abnormal otoscopic examination results, tympanogram compliance ≤ 0.3 ml, or poor-quality results in tympanogram were excluded to avoid analyzing data for conductive or mixed hearing loss. Our analyses were further adjusted for confounding factors that included age, sex, race, education level, BMI, diabetes, hypertension, serum cotinine level, and noise exposure that could result in a misinterpretation. The effects of PUFAs on both low-frequency and high-frequency hearing levels were estimated, with the result of broader frequency estimates than those in previous research studies (11, 12, 14). In addition, individual serum PUFAs were used as a valid estimate of dietary intake of fatty acids (29).

Despite these strengths, this study also has some limitations, which should be mentioned. The results of this study did not permit a temporal relation to be examined because of the cross-sectional design of the NHANES (17). Although the status of serum PUFAs may vary widely depending on dietary intake, this study looked at their concentrations at the one-time point. Furthermore, some potential confounders were not calculated in the models; only the main confounders, which have been reported in previous studies, were included. The results would be more accurate if we consider all other confounders.

## Conclusion

According to the results of the NHANES data analyses, some serum PUFAs were inversely associated with low-frequency PTA, especially in men, while others were positively related to high-frequency PTA, particularly in the 40–59 years old cohort. Furthermore, some of the serum PUFAs were positively associated with both hearing threshold subgroups in women. In general, serum PUFAs tended to be beneficial for low-frequency hearing status and detrimental to the high-frequency hearing threshold. The male sex may play a protective role in this association, while the female sex and middle age may be detrimental in the effect of PUFAs on hearing function.

## Data availability statement

Publicly available datasets were analyzed in this study. This data can be found at: https://wwwn.cdc.gov/nchs/nhanes/continuousnhanes/default.aspx?BeginYear=2011.

## Author contributions

LL, ZJ, and XT completed the conceptualization. LL made a formal analysis of the data, wrote the original draft, and completed the methodology. ZJ and XT completed the review and editing, revising, and final approval and are accountable for all aspects. All authors have approved the final manuscript as submitted.

## Conflict of interest

The authors declare that the research was conducted in the absence of any commercial or financial relationships that could be construed as a potential conflict of interest.

## Publisher's note

All claims expressed in this article are solely those of the authors and do not necessarily represent those of their affiliated organizations, or those of the publisher, the editors and the reviewers. Any product that may be evaluated in this article, or claim that may be made by its manufacturer, is not guaranteed or endorsed by the publisher.
