# Supplementary material for: Serum polyunsaturated fatty acids and hearing threshold shifts in adults in the United States: A cross-sectional study
Source: Front Public Health. 2022 Nov 16;10:939827. doi: 10.3389/fpubh.2022.939827 (PMC9708739; doi:10.3389/fpubh.2022.939827)
Supplement: Supplementary file 1 [file Table_1.DOCX]

Supplementary Table 1 The range of PUFA values for each tertile.

|  | Tertile 1 (umol/L) | Tertile 2 (umol/L) | Tertile 3 (umol/L) |
| --- | --- | --- | --- |
| LA | 1430-3320 | 3330-4010 | 4020-8350 |
| GLA | 5.17-41.4 | 41.5-67.2 | 67.3-322 |
| EDA | 8.75-19.1 | 19.2-25.1 | 25.2-67.7 |
| HGLA | 36.4-129 | 130-176 | 177-465 |
| AA | 187-746 | 747-951 | 952-1880 |
| DTA | 7.21-21.5 | 21.6-29 | 29.1-87.5 |
| DPAn-6 | 3.72-16.3 | 16.4-22.9 | 23-83.3 |
| ALA | 15.2-64 | 64.2-96.4 | 96.5-652 |
| EPA | 8.84-37.5 | 37.7-64 | 64.3-437 |
| DPA | 12.6-41 | 41.1-55.1 | 55.2-142 |
| DHA | 25.7-118 | 119-172 | 173-625 |
