# Supplementary material for: Serum polyunsaturated fatty acids and hearing threshold shifts in adults in the United States: A cross-sectional study
Source: Front Public Health. 2022 Nov 16;10:939827. doi: 10.3389/fpubh.2022.939827 (PMC9708739; doi:10.3389/fpubh.2022.939827)
Supplement: Supplementary file 2 [file Table_2.DOCX]

Supplementary Table 2 Adjusted ^a^ associations between PUFAs and hearing threshold shifts stratified by age (N = 913).

|  |  | LA (umol/L) β (95% CI) | | | *P* _trend_ | *P* _interaction_ |
| --- | --- | --- | --- | --- | --- | --- |
|  |  | Tertile 1 | Tertile 2 | Tertile 3 |  |  |
| **Low-frequency PTA** | 20 ≤ y < 40 | Ref | -0.24 (-1.36, 0.89) | -0.48 (-1.77, 0.81) | 0.4643 | 0.8616 |
|  | 40 ≤ y <60 | Ref | -0.45 (-2.30, 1.40) | 0.18 (-1.71, 2.07) | 0.7994 |  |
|  | 60 ≤ y <69 | Ref | -2.33 (-7.60, 2.94) | -2.02 (-6.83, 2.79) | 0.4497 |  |
| **High-frequency PTA** | 20 ≤ y < 40 | Ref | -0.94 (-2.64, 0.76) | -0.49 (-2.44, 1.45) | 0.5313 | 0.0647 |
|  | 40 ≤ y <60 | Ref | 2.93 (-1.02, 6.89) | 8.00 (3.96, 12.05) | <0.0001 |  |
|  | 60 ≤ y <69 | Ref | -2.99 (-10.61, 4.63) | -2.93 (-9.89, 4.02) | 0.4382 |  |
|  |  | GLA (umol/L) β (95% CI) | | | *P* _trend_ | *P* _interaction_ |
|  |  | Tertile 1 | Tertile 2 | Tertile 3 |  |  |
| **Low-frequency PTA** | 20 ≤ y < 40 | Ref | -0.34 (-1.47, 0.79) | 0.70 (-0.59, 1.99) | 0.3827 | 0.4019 |
|  | 40 ≤ y <60 | Ref | -1.87 (-3.92, 0.18) | -1.71 (-3.65, 0.23) | 0.1602 |  |
|  | 60 ≤ y <69 | Ref | -3.68 (-9.34, 1.98) | -0.59 (-6.10, 4.91) | 0.8150 |  |
| **High-frequency PTA** | 20 ≤ y < 40 | Ref | 0.46 (-1.24, 2.17) | 0.88 (-1.07, 2.83) | 0.3672 | **0.0326** |
|  | 40 ≤ y <60 | Ref | -5.85 (-10.27, -1.44) | 0.55 (-3.62, 4.73) | 0.1695 |  |
|  | 60 ≤ y <69 | Ref | -5.27 (-13.45, 2.91) | -6.43 (-14.39, 1.53) | 0.1465 |  |
|  |  | EDA (umol/L) β (95% CI) | | | *P* _trend_ | *P* _interaction_ |
|  |  | Tertile 1 | Tertile 2 | Tertile 3 |  |  |
| **Low-frequency PTA** | 20 ≤ y < 40 | Ref | 1.08 (-0.06, 2.21) | 0.93 (-0.40, 2.25) | 0.1250 | 0.0909 |
|  | 40 ≤ y <60 | Ref | -0.40 (-2.25, 1.44) | -0.63 (-2.30, 1.04) | 0.4647 |  |
|  | 60 ≤ y <69 | Ref | 0.87 (-4.55, 6.30) | -0.89 (-6.05, 4.27) | 0.6164 |  |
| **High-frequency PTA** | 20 ≤ y < 40 | Ref | 1.65 (-0.06, 3.37) | 1.80 (-0.19, 3.80) | 0.0571 | 0.0679 |
|  | 40 ≤ y <60 | Ref | 2.75 (-1.19, 6.68) | 7.42 (3.85, 10.98) | <0.0001 |  |
|  | 60 ≤ y <69 | Ref | 0.09 (-7.77, 7.96) | 0.14 (-7.34, 7.62) | 0.9718 |  |
|  |  | AA (umol/L) β (95% CI) | | | *P* _trend_ | *P* _interaction_ |
|  |  | Tertile 1 | Tertile 2 | Tertile 3 |  |  |
| **Low-frequency PTA** | 20 ≤ y < 40 | Ref | -1.54 (-2.64, -0.44) | -1.66 (-2.95, -0.38) | 0.0039 | 0.1434 |
|  | 40 ≤ y <60 | Ref | -1.43 (-3.32, 0.45) | -1.28 (-3.16, 0.59) | 0.2425 |  |
|  | 60 ≤ y <69 | Ref | 3.41 (-1.78, 8.60) | -0.15 (-4.90, 4.59) | 0.7320 |  |
| **High-frequency PTA** | 20 ≤ y < 40 | Ref | -0.90 (-2.58, 0.77) | -0.64 (-2.60, 1.33) | 0.4140 | **<0.0001** |
|  | 40 ≤ y <60 | Ref | -0.07 (-4.21, 4.06) | 1.98 (-2.14, 6.10) | 0.2841 |  |
|  | 60 ≤ y <69 | Ref | 0.61 (-6.90, 8.13) | -4.06 (-10.93, 2.80) | 0.1874 |  |
|  |  | ALA (umol/L) β (95% CI) | | | *P* _trend_ | *P* _interaction_ |
|  |  | Tertile 1 | Tertile 2 | Tertile 3 |  |  |
| **Low-frequency PTA** | 20 ≤ y < 40 | Ref | 0.60 (-0.55, 1.75) | 0.79 (-0.44, 2.02) | 0.1978 | 0.2176 |
|  | 40 ≤ y <60 | Ref | -0.23 (-2.08, 1.61) | 0.47 (-1.44, 2.39) | 0.5732 |  |
|  | 60 ≤ y <69 | Ref | 2.67 (-2.30, 7.63) | -1.00 (-5.55, 3.55) | 0.6101 |  |
| **High-frequency PTA** | 20 ≤ y < 40 | Ref | 0.41 (-1.32, 2.15) | 1.77 (-0.08, 3.62) | 0.0669 | 0.1439 |
|  | 40 ≤ y <60 | Ref | -4.74 (-8.63, -0.86) | 4.59 (0.56, 8.62) | 0.0074 |  |
|  | 60 ≤ y <69 | Ref | 0.67 (-6.51, 7.85) | -4.10 (-10.68, 2.47) | 0.2047 |  |
|  |  | DPA (umol/L) β (95% CI) | | | *P* _trend_ | *P* _interaction_ |
|  |  | Tertile 1 | Tertile 2 | Tertile 3 |  |  |
| **Low-frequency PTA** | 20 ≤ y < 40 | Ref | -0.47 (-1.58, 0.65) | -0.79 (-2.20, 0.62) | 0.2479 | 0.9819 |
|  | 40 ≤ y <60 | Ref | -0.03 (-2.15, 2.10) | -1.30 (-3.38, 0.77) | 0.1231 |  |
|  | 60 ≤ y <69 | Ref | -6.29 (-11.92, -0.67) | 0.54 (-4.76, 5.84) | 0.1933 |  |
| **High-frequency PTA** | 20 ≤ y < 40 | Ref | -0.34 (-2.02, 1.35) | -1.02 (-3.15, 1.10) | 0.3598 | **0.0004** |
|  | 40 ≤ y <60 | Ref | -3.97 (-8.60, 0.65) | 0.30 (-4.21, 4.82) | 0.3741 |  |
|  | 60 ≤ y <69 | Ref | -10.80 (-18.93, -2.67) | -1.38 (-9.04, 6.28) | 0.4210 |  |

^a^ Adjusted for age, gender, race/ethnicity, education level, BMI, diabetes, hypertension, serum cotinine level, firearm noise exposure, occupational noise exposure and recreational noise exposure.
