# Supplementary material for: Serum polyunsaturated fatty acids and hearing threshold shifts in adults in the United States: A cross-sectional study
Source: Front Public Health. 2022 Nov 16;10:939827. doi: 10.3389/fpubh.2022.939827 (PMC9708739; doi:10.3389/fpubh.2022.939827)
Supplement: Supplementary file 3 [file Table_3.DOC]

Supplementary Table 3 Adjusted ^a^ associations between PUFAs and hearing threshold shifts stratified by gender (N = 913).

|  |  | LA (umol/L) β (95% CI) | | | *P* _trend_ | *P* _interaction_ |
| --- | --- | --- | --- | --- | --- | --- |
|  |  | Tertile 1 | Tertile 2 | Tertile 3 |  |  |
| **Low-frequency PTA** | Male | Ref | -0.67 (-2.20, 0.86) | -0.97 (-2.60, 0.67) | 0.2445 | **0.0250** |
|  | Female | Ref | 0.06 (-1.51, 1.64) | 0.62 (-1.05, 2.29) | 0.4715 |  |
| **High-frequency PTA** | Male | Ref | 1.16 (-1.98, 4.29) | 2.57 (-0.78, 5.92) | 0.1328 | 0.8441 |
|  | Female | Ref | -1.49 (-3.85, 0.86) | 2.40 (-0.10, 4.90) | 0.0679 |  |
|  |  | GLA (umol/L) β (95% CI) | | | *P* _trend_ | *P* _interaction_ |
|  |  | Tertile 1 | Tertile 2 | Tertile 3 |  |  |
| **Low-frequency PTA** | Male | Ref | -0.51 (-2.18, 1.16) | -0.75 (-2.38, 0.88) | 0.7478 | **0.0217** |
|  | Female | Ref | -1.02 (-2.61, 0.58) | 0.69 (-1.07, 2.45) | **0.0137** |  |
| **High-frequency PTA** | Male | Ref | -2.52 (-5.93, 0.88) | -0.05 (-3.37, 3.27) | 0.2505 | 0.7622 |
|  | Female | Ref | -1.93 (-4.32, 0.47) | 2.18 (-0.47, 4.82) | 0.0001 |  |
|  |  | HGLA (umol/L) β (95% CI) | | | *P* _trend_ | *P* _interaction_ |
|  |  | Tertile 1 | Tertile 2 | Tertile 3 |  |  |
| **Low-frequency PTA** | Male | Ref | 0.62 (-0.99, 2.24) | 0.35 (-1.31, 2.02) | 0.7131 | 0.1253 |
|  | Female | Ref | -0.31 (-2.01, 1.39) | -0.10 (-1.84, 1.63) | 0.9487 |  |
| **High-frequency PTA** | Male | Ref | 1.92 (-1.38, 5.23) | 2.48 (-0.92, 5.88) | 0.1624 | 0.5053 |
|  | Female | Ref | 1.52 (-1.03, 4.07) | 3.23 (0.62, 5.84) | 0.0140 |  |
|  |  | DTA (umol/L) β (95% CI) | | | *P* _trend_ | *P* _interaction_ |
|  |  | Tertile 1 | Tertile 2 | Tertile 3 |  |  |
| **Low-frequency PTA** | Male | Ref | -0.70 (-2.32, 0.92) | -1.55 (-3.21, 0.11) | 0.0681 | **0.0169** |
|  | Female | Ref | -1.13 (-2.70, 0.44) | 0.94 (-0.77, 2.65) | 0.2813 |  |
| **High-frequency PTA** | Male | Ref | -0.09 (-3.40, 3.23) | -1.79 (-5.20, 1.62) | 0.3035 | 0.5373 |
|  | Female | Ref | -1.67 (-4.03, 0.69) | 2.93 (0.37, 5.49) | 0.0253 |  |
|  |  | EPA (umol/L) β (95% CI) | | | *P* _trend_ | *P* _interaction_ |
|  |  | Tertile 1 | Tertile 2 | Tertile 3 |  |  |
| **Low-frequency PTA** | Male | Ref | -0.03 (-1.65, 1.58) | -1.14 (-2.82, 0.54) | 0.1793 | 0.0713 |
|  | Female | Ref | -1.46 (-3.20, 0.28) | -0.87 (-2.68, 0.94) | 0.4196 |  |
| **High-frequency PTA** | Male | Ref | -0.71 (-4.02, 2.60) | 2.61 (-0.82, 6.05) | 0.1324 | 0.2381 |
|  | Female | Ref | -1.21 (-3.82, 1.40) | 1.23 (-1.48, 3.94) | 0.2934 |  |
|  |  | DPA (umol/L) β (95% CI) | | | *P* _trend_ | *P* _interaction_ |
|  |  | Tertile 1 | Tertile 2 | Tertile 3 |  |  |
| **Low-frequency PTA** | Male | Ref | -0.90 (-2.59, 0.80) | -1.59 (-3.58, 0.41) | 0.1200 | **0.0012** |
|  | Female | Ref | -0.72 (-2.42, 0.98) | 1.14 (-0.88, 3.15) | 0.2709 |  |
| **High-frequency PTA** | Male | Ref | -4.12 (-7.59, -0.66) | -1.77 (-5.85, 2.31) | 0.4296 | 0.5709 |
|  | Female | Ref | -1.70 (-4.24, 0.84) | 2.28 (-0.72, 5.29) | 0.1396 |  |

^a^ Adjusted for age, race/ethnicity, education level, BMI, diabetes, hypertension, serum cotinine level, firearm noise exposure, occupational noise exposure and recreational noise exposure.
